# Suppression of reproductive characteristics of the invasive plant Mikania micrantha by sweet potato competition

**DOI:** 10.1186/s12898-016-0085-9

**Published:** 2016-06-20

**Authors:** Shicai Shen, Gaofeng Xu, David Roy Clements, Guimei Jin, Shufang Liu, Yanxian Yang, Aidong Chen, Fudou Zhang, Hisashi Kato-Noguchi

**Affiliations:** Agricultural Environment and Resource Research Institute, Yunnan Academy of Agricultural Sciences, Kunming, 650205 Yunnan China; Biology Department, Trinity Western University, 7600 Glover Road, Langley, BC V2Y1Y1 Canada; Department of Applied Biological Science, Faculty of Agriculture, Kagawa University, Miki, Kagawa 761-0795 Japan

**Keywords:** Biological control, *Mikania micrantha*, Sweet potato, Competition, Flowering and fruiting phenology, Reproductive suppression

## Abstract

**Background:**

As a means of biologically controlling *Mikania micrantha* H.B.K. in Yunnan, China, the influence of sweet potato [*Ipomoea batatas* (L.) Lam.] on its reproductive characteristics was studied. The trial utilized a de Wit replacement series incorporating six ratios of sweet potato and *M. micrantha* plants in 25 m^2^ plots over 2 years.

**Results:**

Budding of *M. micrantha* occurred at the end of September; flowering and fruiting occurred from October to February. Flowering phenology of *M. micrantha* was delayed (P < 0.05), duration of flowering and fruiting was reduced (P < 0.05) and duration of bud formation was increased (P < 0.05) with increasing proportions of sweet potato. Reproductive allocation, reproductive investment and reproductive index of *M. micrantha* were significantly reduced (P < 0.05) with increasing sweet potato densities. Apidae bees, and Calliphoridae or Syrphidae flies were the most abundant visitors to *M. micrantha* flowers. Overall flower visits decreased (P < 0.05) as sweet potato increased. Thus the mechanism by which sweet potato suppressed sexual reproduction in *M. micrantha* was essentially two-fold: causing a delay in flowering phenology and reducing pollinator visits. The number, biomass, length, set rate, germination rate, and 1000-grain dry weight of *M. micrantha* seeds were suppressed (P < 0.05) by sweet potato competition. With proportional increases in sweet potato, sexual and asexual seedling populations of *M. micrantha* were significantly reduced (P < 0.05). The mortality of both seedling types increased (P < 0.05) with proportional increases in sweet potato.

**Conclusions:**

These results suggest that sweet potato significantly suppresses the reproductive ability of the invasive species *M. micrantha*, and is a promising alternative to traditional biological control and other methods of control. Planting sweet potato in conjunction with other control methods could provide a comprehensive strategy for managing *M. micrantha*. The scenario of controlling *M. micrantha* by utilizing a crop with a similar growth form may provide a useful model for similar management strategies in other systems.

## Background

Plant invasions have received attention on a global scale. Invasive plants have caused great economic harm, biodiversity loss, environmental problems, and even human and animal health issues [1, 2]. Currently, methods to control invasive plants have been widely investigated and practiced, including mechanical [3–5], chemical [6, 7] and biological control [8–11]. However, mechanical measures may potentially accelerate invasions [3], chemical measures can be detrimental to non-target species and environment health [12–14], and traditional biological control measures via introduction of pathogens, parasites, and predators against invaders are expensive and pose risks to ecosystem integrity [15, 16]. Thus, as a potential alternative to traditional biological control which generally employs insects or pathogens, replacement control relies on growth characteristics of one or more plants to suppress exotic plants, simultaneously reducing damage caused by the invasive species and improving local natural ecosystem health by reducing the potential for invasive plants to spread beyond agricultural fields. Adoption of this alternative method has received considerable attention in recent years [17–21].

*Mikania micrantha* H.B.K. is a rapidly-growing perennial creeping vine belonging to the family Asteraceae native to Central and South America [22]. The vine has been listed among the top 100 worst invasive species [22, 23] and as one of the top 10 worst weeds in the world [22]. *M. micrantha* is present in tropical Asia, parts of Papua New Guinea, Indian Ocean islands, Pacific Ocean Islands, and Florida in the US [22, 24, 25]. It has colonized a broad range of farming systems and forest lands, banks of streams and rivers, roadsides and railway tracks, pastures, and open disturbed areas [22], leading to serious economic loss, biodiversity loss and negative environmental impacts [7, 18, 22, 26, 27].

To explore ecological methods for managing *M. micrantha*, biological control measures through replacement control with high value species (e.g., local food, native species and/or cash crops) have been investigated [18, 28–31]. In 2006 and 2007, sweet potato [*Ipomoea batatas* (L.) Lam.: Convolvulaceae], an important locally grown cash crop native to the American tropics, was observed to inhibit *M. micrantha* growth in Longchuan County of Yunnan Province, China in sweet potato fields where *M. micrantha* occurred [30]. Subsequent studies examined the effects of a local crop, sweet potato, on *M. micrantha* growth and soil nutrients [18]. Sweet potato exhibited greater competitive ability than *M. micrantha*, with plant height, branch, leaf, stem node, adventitious root, and biomass of *M. micrantha* suppressed significantly; furthermore sweet potato also demonstrated higher levels of nutrient uptake than *M. micrantha*. Moreover, flowering of *M. micrantha* was significantly suppressed in mixed culture with sweet potato and with decreasing proportions of *M. micrantha*, the competitiveness of sweet potato increased at a rate exceeding what would be predicted by the increase in relative density [18]. However, no literature is available on the effects of sweet potato competition on the entire suite of reproductive characteristics of *M. micrantha*.

Building on our former studies [18, 30, 31], the present research examined how sweet potato suppressed reproductive characteristics of *M. micrantha* in Yunnan Province, China. Previous reports did not refer to impacts on reproductive characteristics, so this is the first report of how sweet potato competition affects characteristics such as flowering and seed production in *M. micrantha*. These findings are important to further elucidate the competitive interaction and mechanisms between sweet potato and *M. micrantha* and provide insights for similar ecological control methods that could be applied to other invasive alien species.

## Methods

### Study site

The study site was located in Longchuan County (24°08′–24°39′ N, 97°17′–97°39′ E), in the western end of Yunnan Province, Southwest China. This area is characterized by a typical tropical climate, having a rainy season featuring heavy rainfall with 90 % relative humidity alternating with a dry season [30]. Rainfall averages 15450 mm per year and the annual mean temperature is 18.9 °C. Recently, the range of *M. micrantha* has been expanding rapidly within Longchuan County, as the plant has invaded agricultural areas and forest margins [7].

### Study species

*Mikania micrantha* is one of the most serious invasive species in Longchuan County where this study took place. This perennial weed exhibits a climbing growth form in forests, orchards and shrublands, but on roadsides, in open wasteland areas without crops, and other areas without woody vegetation, it takes on a prostrate form. It has infested sugarcane, orange, banana, coffee, pineapple, bamboo, sweet potato, maize crops, as well as artificial pasture and secondary forest in the study area [7]. *M. micrantha* can invade disturbed environments via light weight wind-dispersed seeds that are produced in great numbers, as high as 170,000 m^−2^ [32]. At a local level, vegetative reproduction is responsible for most population growth as facilitated by rooting of stem fragments [3].

Sweet potato, native to the American tropics, is one of the main food and cash crops in tropical and subtropical regions of Yunnan Province. It is also grown in many other regions of China and other subtropical or warm-temperate regions of the world as a food source. In Longchuan County, local villagers have grown it for over 100 years [30]. This herbaceous perennial vine usually exhibits a prostrate growth form in agricultural areas, so its niche is similar to that of *M. micrantha*. Because of its purple root, it is also known as purple sweet potato. The aboveground parts of the plant are used for livestock fodder and its roots are used for human eating. It is propagated by seed or by clonal means, with 20–50 cm fragments with 3–5 nodes typically planted [33].

### Experiment design and data collection

The experiments were conducted during the May, 2014-October, 2015 growing season within maize and sweet potato intercropping land in the vicinity of Zhangfeng Town, Longchuan County, utilizing a de Wit replacement series method [34]. On 7 May 2014, whole *M. micrantha* plants (including roots) were collected from a *M. micrantha* population located in a nearby forest margin and whole sweet potato plants were collected from farmland, respectively. To ensure relative uniformity among the experimental stock, one-node segments (fresh weight 3.0–3.5 g, 7–8 cm pieces) were taken from central stem portions of relatively young plants of similar size from both species. The segments were placed in Hoagland’s solution [35] and grown for 10 days. On 17 May 2014, the sprouts derived from cuttings of both species were transplanted in the field test plots. Six ratios of sweet potato and *M. micrantha* plants were utilized (3:1, 2:1, 1:1, 1:2, 1:3, 0:4) while maintaining a constant planting density of 20 plants m^−2^ (0.25 × 0.20 m spacing). All plots were arranged in a complete randomized design with 4 replicates utilizing 25 m^2^ plots (5 × 5 m). All sweet potato and *M. micrantha* plants were distributed evenly within the plot. During the experiment, the two species exhibited prostrate growth. The plots were not weeded and no fertilizers were used.

From October 2014 to February 2015, flowering and fruiting phenology were recorded at 3 days intervals for *M. micrantha*, including the dates of initial and last budding, flowering, fruiting and seed set. During peak flowering times, we marked 20 inflorescences of *M. micrantha* in each plot and recorded the number of flower visits per insect visitor on each inflorescence between 9:00 and 18:00 (time of maximum pollinator activity) for two continuous days with all plots monitored simultaneously over the same two days. According to [36] each flower visitor was classified as belonging to Apidae, Calliphoridae, Syrphidae (generally the most frequent visitors to *M. micrantha*), or another pollinator group beyond these three taxa. At the same time, twenty plants of each species were selected randomly and harvested within the middle region of each plot. *M. micrantha* plants were carefully removed and separated, and number of inflorescences and flowers were recorded. The fresh weight of inflorescences and flowers and total biomass of each *M. micrantha* plant were measured. On 28 January 2015, seed production of *M. micrantha* was measured in the study plots after flowering had waned, but prior to seed dispersal. Another twenty plants of each species were selected randomly and harvested within the middle region of each plot. The number, size (length) and dry weight of *M. micrantha* seeds were measured. Sixty days later, germination rates of *M. micrantha* seeds from each plot were tested in the laboratory.

During the spring, summer and fall of 2015 (March–October), four small quadrats (1 × 1 m) were selected randomly and marked in each plot. Seedlings were identified as either produced from germinating seeds (sexual) or vegetative growth (asexual). The number of new sexual and asexual *M. micrantha* seedlings was monitored in each quadrat monthly. We did not remove the seedlings that were counted but rather kept track of the total that emerged through the season, month by month. Seedling mortality was also recorded for the *M. micrantha* that emerged.

### Data analyses

Reproductive characteristics of *M. micrantha* [37, 38] were calculated in each plot with the following parameters: (1) Reproductive allocation (g·g^−1^) = inflorescence biomass/total biomass of each plant, (2) Reproductive investment (g·g^−1^) = flower biomass/total biomass of each plant, (3) Reproductive index (g·g^−1^) = flower biomass/inflorescence biomass of each plant, (4) Reproductive ratio (flower·mg^−1^) = flower number/inflorescence biomass of each plant and (5) Reproductive efficiency index (flower·mg^−1^) = flower number/total biomass of each plant.

All growth variables (flowering, bud formation and fruiting duration, inflorescence number, flower number, germination, and biomass of inflorescences, and flowers and seeds) of *M. micrantha* plants were analyzed by analysis of variance (one-way ANOVA) using IBM SPSS 22.0 software. If significant differences were detected with the ANOVA, Tukey’s HSD, Post Hoc Multiple Comparisons, Homogeneity of Variance tests were used to detect differences among treatments at a 5 % level of significance.

## Results

### Reproductive phenology of *Mikania micrantha*

Budding of *M. micrantha* occurred at the end of September, and flowering and fruiting occurred from October to February in our study area (Table 1). The peak bloom occurred between mid-November and early December. Fruiting started as early as the end of November, and almost all fruits had dropped by early February. The flowers opened throughout the day, but the majority did so in the morning. In monoculture, the duration of bud formation, flowers and fruits of *M. micrantha* was 27.25 ± 0.05 d, 79.75 ± 1.71 d, and 75.75 ± 1.71 d, respectively (Table 1). With increased sweet potato: *M. micrantha* ratios, the initial date of budding, flowering and fruiting of *M. micrantha* was significantly delayed, and the duration of flowering and fruiting of *M. micrantha* significantly declined, but duration of bud formation of *M. micrantha* was markedly increased (P < 0.05).

**Table 1:** Flowering and fruiting phenology of *Mikania micrantha* growing as a monoculture or under mixed culture conditions

Variables	Ratios (sweet potato: *M. micrantha*)					
	3:1	2:1	1:1	1:2	1:3	0:4
Initial budding date	10 October	5 October	2 October	28 September	26 September	26 September
Initial flowering date	16 November	12 November	4 November	27 October	24 October	24 October
Initial fruiting date	20 November	16 November	11 November	4 November	2 November	1 November
Duration of bud formation (d)	37.75 ± 0.96a	34.75 ± 0.96b	31.25 ± 0.96c	30.25 ± 0.96c	28.25 ± 0.96 cd	27.25 ± 0.05d
Duration of flowering (d)	48.25 ± 1.26f	54.75 ± 1.50e	60.50 ± 1.29d	68.25 ± 2.22c	73.75 ± 2.06b	79.75 ± 1.71a
Duration of fruiting (d)	48.25 ± 1.89e	55.00 ± 2.16d	59.50 ± 1.29c	66.50 ± 1.29b	70.00 ± 1.63b	75.75 ± 1.71a

Data are expressed as mean ± standard deviation. The different letters within same row signify significantly different at P < 0.05

A total of about 47 flower visits of *M. micrantha* were observed for each inflorescence per day, with Apidae (bees), Calliphoridae (flies), Syrphidae (flies) and other pollinators accounting for 61.2, 22.0, 12.4, and 4.4 % of total visits, respectively, in monoculture (Table 2). Overall flower visiting behavior and visitation rate of Apidae, Calliphoridae and Syrphidae per inflorescence were substantially reduced with increasing proportions of sweet potato (P < 0.05).

**Table 2:** Total number of visits (visits per day and inflorescence) by the four pollinator groups to *Mikania micrantha* growing as a monoculture or under mixed culture conditions

Pollinators	Ratios (sweet potato: *M. micrantha*)					
	3:1	2:1	1:1	1:2	1:3	0:4
Apidae	8.75 ± 0.95d	10.50 ± 1.11d	13.50 ± 1.04c	24.50 ± 1.14b	27.50 ± 1.43a	28.50 ± 1.40a
Calliphoridae	4.92 ± 0.70c	6.00 ± 0.63c	6.50 ± 0.50bc	8.02 ± 0.75b	9.74 ± 0.88a	10.26 ± 0.96a
Syrphidae	1.26 ± 0.46c	1.54 ± 0.45c	2.28 ± 0.40c	3.76 ± 0.39b	5.63 ± 0.54a	5.75 ± 0.85a
Other pollinator	2.53 ± 0.48a	1.63 ± 0.57a	1.76 ± 0.41a	2.23 ± 0.73a	1.77 ± 0.92a	2.04 ± 0.67a
Total	17.46 ± 1.19d	19.66 ± 2.60d	24.04 ± 1.76c	38.50 ± 1.55b	44.63 ± 2.13a	46.55 ± 0.95a

Data are expressed as mean ± standard deviation. Different letters within the same row signify significant differences at P < 0.05

### Reproductive characteristics of *Mikania micrantha*

In monoculture, the biomass of plant, inflorescences and flowers of *M. micrantha* was 301.61 ± 5.19 g, 54.66 ± 1.14 g, and 40.99 ± 1.01 g, respectively; the inflorescence and flower numbers of *M. micrantha* were 2647.8 ± 55.3 and 10,587.7 ± 239.3, respectively. In mixed culture, the total biomass of plant, biomass of inflorescences and flowers, and numbers of inflorescences and flowers of *M. micrantha* were significantly (P < 0.05) suppressed with decreasing proportions of *M. micrantha* (Table 3). With proportional increases in sweet potato, reproductive allocation, reproductive investment and reproductive index of *M. micrantha* were significantly lower (P < 0.05). The reproductive ratio of *M. micrantha* did not differ significantly among treatments (Table 3). With decreasing proportions of *M. micrantha*, the reproductive efficiency index of *M. micrantha* was reduced to a certain extent by sweet potato but the trend was not clear.

**Table 3:** Flowering characteristics of *Mikania micrantha* growing as a monoculture or under mixed culture conditions

Variables	Ratios (sweet potato: *M. micrantha*)					
	3:1	2:1	1:1	1:2	1:3	0:4
Total biomass (g)	25.16 ± 1.10f	35.88 ± 0.96e	54.80 ± 0.80d	82.21 ± 1.36c	104.41 ± 2.62b	301.61 ± 5.19a
Flower number	429.9 ± 10.3f	1168.3 ± 82.7e	2486.2 ± 62.9d	3508.8 ± 53.8c	4705.9 ± 106.5b	10,587.7 ± 239.3a
Inflorescence number	108.3 ± 3.7f	289.8 ± 14.1e	613.9 ± 12.1d	868.8 ± 16.1c	1179.4 ± 22.5b	2647.8 ± 55.3a
Flower biomass (g)	0.63 ± 0.03e	1.55 ± 0.04e	3.86 ± 0.06d	7.32 ± 0.06c	11.39 ± 0.29b	40.99 ± 1.01a
Inflorescence biomass (g)	1.24 ± 0.04e	2.28 ± 0.07e	6.86 ± 0.09d	11.21 ± 0.13c	14.95 ± 0.27b	54.66 ± 1.14a
Reproductive allocation (g·g^−1^)	0.049 ± 0.004f	0.064 ± 0.002e	0.125 ± 0.002d	0.136 ± 0.001c	0.143 ± 0.002b	0.181 ± 0.002a
Reproductive investment (g·g^−1^)	0.025 ± 0.002f	0.043 ± 0.001e	0.071 ± 0.001d	0.089 ± 0.002c	0.109 ± 0.001b	0.136 ± 0.002a
Reproductive index (g·g^−1^)	0.512 ± 0.012d	0.678 ± 0.005b	0.563 ± 0.003c	0.653 ± 0.010b	0.762 ± 0.012a	0.750 ± 0.018a
Reproductive ratio (flower·mg^−1^)	3.970 ± 0.081a	4.028 ± 0.089a	4.049 ± 0.025a	4.039 ± 0.025a	3.990 ± 0.015a	3.999 ± 0.041a
Reproductive efficiency index (flower·mg^−1^)	0.017 ± 0.001d	0.033 ± 0.002c	0.045 ± 0.001a	0.043 ± 0.000b	0.045 ± 0.001ab	0.035 ± 0.000c

Data are expressed as mean ± standard deviation. The different letters within same row signify significant differences at P < 0.05

For a ratio of sweet potato to *M. micrantha* of 3:1, the number and biomass of *M. micrantha* seeds were reduced by a factor of more than 100 compared to *M. micrantha* in monoculture, i.e., 17,632.6 ± 479.8 vs. 171.7 ± 4.3 and 1.772 ± 0.042 g vs. 0.014 ± 0.000 g, respectively (Table 4). The number, biomass, length, set rate, germination rate, and 1000-grain dry weight of *M. micrantha* seeds were significantly suppressed (P < 0.05) with decreasing proportions of *M. micrantha*.

**Table 4:** Characteristics of *Mikania micrantha* seed growing as a monoculture or under mixed culture conditions

Variables	Ratios (sweet potato: *M. micrantha*)					
	3:1	2:1	1:1	1:2	1:3	0:4
Seed number	171.7 ± 4.3f	702.9 ± 8.2e	1995.1 ± 54.9d	4066.9 ± 94.8c	6797.6 ± 92.2b	17,632.6 ± 479.8a
Seed biomass (g)	0.014 ± 0.000f	0.058 ± 0.001e	0.171 ± 0.004d	0.362 ± 0.008c	0.654 ± 0.011b	1.772 ± 0.042a
Seed length (mm)	0.750 ± 0.008f	0.808 ± 0.013e	0.860 ± 0.008d	0.900 ± 0.012c	1.058 ± 0.013b	1.290 ± 0.014a
Seed set rate (%)	7.99 ± 0.26f	12.07 ± 0.76e	16.05 ± 0.13d	23.18 ± 0.27c	28.90 ± 0.53b	33.31 ± 0.64a
Germination rate (%)	16.88 ± 0.48f	20.63 ± 0.63e	25.13 ± 0.85d	32.63 ± 1.65c	53.13 ± 1.60b	68.25 ± 1.04a
1000-grain dry weight (g)	0.078 ± 0.001f	0.082 ± 0.001e	0.086 ± 0.001d	0.089 ± 0.001c	0.096 ± 0.001b	0.101 ± 0.001a

Data are expressed as mean ± standard deviation. Different letters within the same row signify significant differences at P < 0.05

### Seedling population dynamics of *Mikania micrantha*

Sexual seedling populations of *M. micrantha* germinated for 6 months (March–August), primarily occurring between May–June. Asexual seedling populations first arose in March, and then increased in density monthly from March to October. In monoculture, sexual population and asexual population densities were 89.25 ± 4.35 m^−2^ and 134.75 ± 4.99 m^−2^, respectively (Table 5). During growth some seedlings died; the sexual seedlings mostly died from May–June, and asexual seedlings did so between July–September. In all treatments, the asexual seedling population was comprised significantly higher densities (P < 0.05) than the sexual seedling population, and the asexual mortality rate was much lower (P < 0.05) than sexual mortality rate. With proportional increases in sweet potato, the total population, sexual population and asexual population of *M. micrantha* significantly declined (P < 0.05); higher seedling mortality rates were also associated with greater proportions of sweet potato.

**Table 5:** Population densities m^−2^ from sexual and asexual reproduction of *Mikania micrantha* growing as a monoculture or under mixed culture conditions

Variables	Ratios (sweet potato: *M. micrantha*)					
	3:1	2:1	1:1	1:2	1:3	0:4
Total population	42.00 ± 2.83f	65.25 ± 2.99e	102.50 ± 3.70d	166.75 ± 6.02c	191.50 ± 4.51b	224.00 ± 6.06a
Sexual population	9.25 ± 1.71f	21.00 ± 2.16e	31.50 ± 2.08d	55.75 ± 3.30c	70.50 ± 1.29b	89.25 ± 4.35a
Asexual population	32.75 ± 1.71f	44.25 ± 2.63e	71.00 ± 2.83d	111.00 ± 3.92c	121.00 ± 3.37b	134.75 ± 4.99a
Sexual mortality rate (%)	0.629 ± 0.092a	0.596 ± 0.080a	0.438 ± 0.036b	0.340 ± 0.035bc	0.291 ± 0.022c	0.241 ± 0.019c
Asexual mortality rate (%)	0.358 ± 0.034a	0.265 ± 0.008b	0.152 ± 0.019c	0.077 ± 0.014d	0.056 ± 0.008de	0.030 ± 0.001e

Data are expressed as mean ± standard deviation. The different letters within same row signify significant differences at P < 0.05

## Discussion

Our research showed that the biomass, flowering, seed and seedling characteristics of *M. micrantha* were reduced when grown in association with sweet potato. A previous study showed that plant height, branch, leaf, stem node, adventitious root, and biomass of *M. micrantha* were suppressed significantly by sweet potato competition [18]. The present study found that the biomass of plant, inflorescences, flowers, and seeds of *M. micrantha* were also significantly suppressed with increasing proportions of sweet potato. Moreover, with decreasing number and biomass of inflorescences and flowers, the reproductive allocation, reproductive investment and reproductive index of *M. micrantha* were also significantly reduced. The net result of the presence of sweet potato was reduced reproductive potential of M*. micrantha* and like other invasive species, its reproductive ability, including flowering characteristics, seed dispersal and seed germination parameters, is associated with its invasiveness [39–43].

Flowering phenology is affected by number, timing and duration of flowers [44]. These factors are not only constrained by genetics and phylogeny, but also affected by environmental conditions, such as sunlight, temperature, nutrients, and competition [42]. The present study found that flowering phenology of *M. micrantha* was significantly delayed, duration of flowering and fruiting was significantly reduced and duration of bud formation was markedly increased with increased sweet potato proportions.

Along with reduced biomass and delayed flowering, another major factor reducing reproductive output in *M. micrantha* observed in our study was the negative impact of sweet potato on pollinator visitation. *M. micrantha* depends on insects for sexual reproduction as it is self-incompatible [45]. For insect-pollinated plant species, competition for pollinators may lead to changes in visitation frequency or pollinator composition [46–48] and consequently, a lowered reproductive output [48]. In this study, species of Apidae (bee species), Calliphoridae (flies), and Syrphidae (flies) were the most abundant visitors to *M. micrantha* flowers and were observed to have the longest foraging time of all floral visitors, which is consistent with the results of other studies [36, 49]. Overall flower visits of Apidae, Calliphoridae and Syrphidae per inflorescence were reduced significantly with increasing proportions of sweet potato. From October to February in the study area, the temperature gradually became lower and the delayed flowering phenology of *M. micrantha* corresponded with reduced insect activity. Moreover, in mixed culture, 70–90 % of *M. micrantha* stems and leaves were covered by sweet potato [18], thus reducing insect visitation via diminished visibility of *M. micrantha* flowers. Because flowering in both sweet potato and *M. micrantha* occurs at virtually the same time, pollinators visited both species during the monitoring period; however, because the number of flowers per shoot was at least 15 times greater for *M. micrantha* than sweet potato [18], the main influence on pollinator visitation was *M. micrantha* flower number. *M. micrantha* can produce a large number of flowers and small, light, and wind-dispersed seeds [32]. The negative correlation we observed between seed set and pollinator visitation in *M. micrantha* is consistent with the commonly observed link between pollinator visitation rate and seed set [50].

Nutrient availability also influences reproductive output by *M. micrantha*, with fewer flowers, lower seed setting percentage, lower 1000-grain weight and shorter flowering duration observed in plants growing in nutrient-deficient soils with suboptimal fertility, but soils with an overabundance of nutrients (e.g., silt from ponds or dump sites) also resulted in fewer flowers and low seed set [51]. Meanwhile, plants growing in an open habitat had more flowers with longer flowering duration, and under shade the 1000-grain weight was shown to have a slight increase, but light that was too bright or too dim was not conducive to seed set [51, 52]. Light was found to affect fruiting; for example, a photoperiod of 12 h/day resulted in 68.4 % of flowers producing fruit [53]. The present study found that the number, biomass, length, set rate, germination rate, and 1000-grain dry weight of *M. micrantha* seeds was significantly suppressed with decreasing proportions of *M. micrantha*. This is because *M. micrantha* plants covered by dense carpets of sweet potato received fewer pollinator visits and produced fewer seeds. Furthermore, sweet potato exhibited greater absorption of soil nutrients than *M. micrantha* [18]; the resulting lack of nutrients likely lead to reduced 1000-grain weight and germination rate of *M. micrantha* in the presence of sweet potato.

The potential for high levels of sexual and/or vegetative reproduction by *M. micrantha* is formidable [22]. It has transient soil seed bank and persistent soil seed bank, and some seeds would germinate given ideal germination conditions such as season, temperature, moisture; otherwise the seeds would remain dormant [54, 55]. Large numbers of seeds of *M. micrantha* were concentrated primarily in the 0–5 cm soil layer, which contained 98 % of the total seeds present in the soil [55]. Vegetative propagation of *M. micrantha* from stem fragments that root easily at the nodes and from vegetative ramets arising from rosettes can be considered at least as important as reproduction by seeds [22, 56]. The seedling is the most vulnerable stage in the life history of *M. micrantha* and seedlings suffer a high level of mortality under natural conditions [54]. The present study found that with proportional increases in sweet potato, both sexual and asexual seedling populations of *M. micrantha* were significantly suppressed, corresponding to increased mortality with increasing levels of sweet potato competition. Thus, the best time to control *M. micrantha* is during the seedling period and control measures should be comprehensive involving both herbicides and appropriate cultural techniques [31].

## Conclusion

The competitive advantage of sweet potato over *M. micrantha* could be used to reduce *M. micrantha* growth and reproductive ability in tropical and subtropical agricultural regions suitable for cultivation of sweet potato. Both plants have similar growth forms and climatic requirements, and sweet potato is a high value crop, and thus we recommend planting sweet potato in areas infested by *M. micrantha*, perhaps as part of a rotation involving more vulnerable crops. Sweet potato could even be planted in habitats such as waste areas not currently cultivated in order to reduce *M. micrantha* populations. Our results showed that various components of reproduction for *M. micrantha* were significantly reduced by suppression of plant growth; the original data is available online [57]. Flowering phenology was impacted by sweet potato competition, and delayed flowering phenology, reduced duration of flowering and fruiting and increased duration of bud formation resulted in reduced pollinator visits and seed set for *M. micrantha*. Finally, high cover of sweet potato shading *M. micrantha* plants also reduced pollinator visits, seeds number, and seedling populations of *M. micrantha*. Thus the mechanism by which sweet potato reduced sexual reproduction in *M. micrantha* was essentially twofold: causing a delay in flowering phenology and reducing pollinator visits. In addition to utilizing sweet potato, research in this study and other recent studies revealed that control of *M. micrantha* ideally should take place during the seedling period when *M. micrantha* is most vulnerable and should be comprehensive for optimal results, employing both chemical and cultural control. Thus in the case of our study region in southern Asia, the most effective timing of control is in the peak of sexual seedling emergence in May–June. The potential for utilizing a crop like sweet potato to compete with an invasive plant may well apply to many other agronomic settings where other management techniques (e.g., chemical control, mechanical control or classical biological control) are unreliable or are associated with environmental concerns. The scenario of controlling *M. micrantha* by utilizing a crop with a similar growth form may provide a useful model for similar management strategies in other systems.
